# Paediatric and perinatal postmortem imaging: the need for a subspecialty approach

**DOI:** 10.1007/s00247-014-3132-8

**Published:** 2014-08-30

**Authors:** Owen J. Arthurs, Rick R. van Rijn, Andrew M. Taylor, Neil J. Sebire

**Affiliations:** 1 Department of Radiology, Great Ormond Street Hospital for Children NHS Foundation Trust, London, WC1N 3JH UK; 2 Department of Radiology, Emma Children’s Hospital - Academic Medical Centre, Amsterdam, The Netherlands; 3 Cardiorespiratory Unit, Great Ormond Street Hospital for Children, London, UK; 4 Department of Pathology, Great Ormond Street Hospital for Children, London, UK; 5 UCL Institute of Child Health, London, UK; 6 Centre for Cardiovascular Imaging, UCL Institute of Cardiovascular Science, London, UK

**Keywords:** Autopsy, Postmortem, MRI, Children, Perinatal, Foetal

## Abstract

Paediatric postmortem imaging is distinct and different from adult postmortem imaging due to differences in disease aetiology, pathology and imaging approaches, which require a particular skill set to maximise its yield and clinical utility. Practitioners need to have expertise in several aspects of radiology, including both plain radiographs and cross-sectional imaging modalities, knowledge of specialist techniques, and familiarity with the unique range of pathologies in this patient population, including perinatal pathology. Here we outline the training requirements that should be considered to establish such a service.

## Introduction

In our opinion, paediatric postmortem imaging is very different from adult postmortem imaging, due to differences in disease aetiology, pathology and imaging approaches, which require a particular skill set to maximise its yield and clinical utility. Practitioners need to have combined expertise in several aspects of radiology, including both plain radiographs and cross-sectional imaging modalities, together with a knowledge of specialist techniques and a familiarity with the unique range of pathologies in this patient population, including perinatal pathology. Here we outline the training requirements that should be considered to establish such a service. Note that throughout this manuscript, we use internationally agreed terminology for postmortem imaging [1].

## What is the clinical need?

The numbers of foetal, stillborn and infant deaths in any country are relatively small in comparison to overall adult deaths (around 1–2% of total United Kingdom deaths are stillbirths, infants and children), but this group still represents a significant clinical issue. This article discusses only the impact of paediatric postmortem imaging, acknowledging the importance of this small group on parental bereavement, genetic counselling and planning of future pregnancies.

**Fig. 1:**
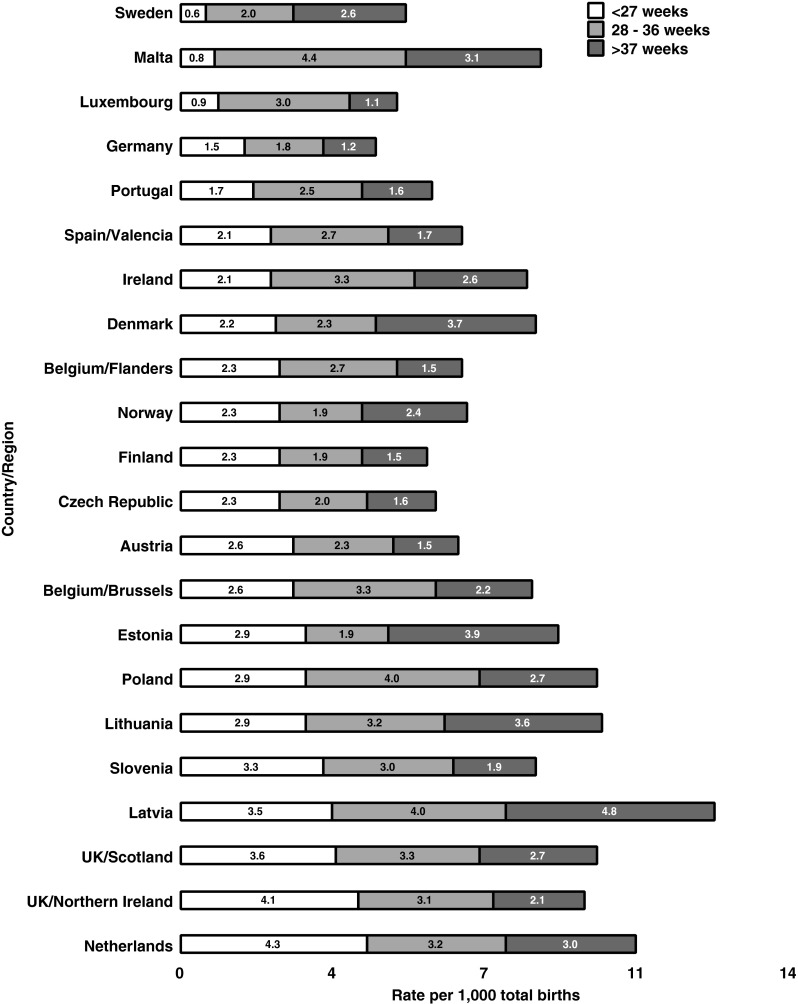
Perinatal mortality rates by European country. Adapted with permission from de Jonge et al., 2013 [2]

The perinatal mortality rate (PMR) is defined as the death of a foetus >24 weeks or early neonatal death <7 days per 1,000 live births (Table 1). Even allowing for slightly different definitions, PMR shows a significant variability across Europe, ranging from 4.6 per 1,000 in Germany to 12.35 per 1,000 in Latvia (2004 data; Fig. 1 [2]). For full-term live births these figures are lower, ranging from 1.2 per 1,000 in Luxembourg to 5.1 per 1,000 in Latvia [2]. This equates to around 36,000 annual perinatal deaths across Europe. In addition, there are a significant number of deaths in infants and children, with many remaining unexplained (Sudden Unexplained Deaths in Infancy; SUDI).

**Table 1:** Definition of types of paediatric death

Term	Definition
Late foetal loss	Delivered showing no signs of life between 20 + 0 and 23 + 6 weeks of pregnancy.
Termination of pregnancy	Induced delivery, with or without fetocide.
Stillbirth	Delivered showing no signs of life after 24 + 0 weeks of pregnancy.
Neonatal death	Death of a live born baby occurring within 28 days of birth.
Early neonatal death	Death of a live born baby occurring within 7 days of birth.
Perinatal death	Stillbirths and early neonatal deaths.
Post-neonatal or infant deaths	Death occurring from the 28th day to 1 year of age.
Childhood deaths	Death between ages 1 year and 16 or 18 years.

Whilst a high percentage of parents indicate a strong desire to know why their child died and that an autopsy helped them cope with their grief, bereaved parents may be reluctant to agree to a full traditional autopsy [3, 4]. It is still perceived to be invasive and unacceptable by many, although there are a variety of reasons for parents’ refusal [3, 4].

Overall autopsy rates have shown a significant decline across the world [5] with neonatal and infant autopsy rates currently at around 30% or lower [6]. This is well below national standard recommendations of 75% and means that large amounts of information that could be used to counsel parents about future pregnancies, and contribute to epidemiological studies regarding infant deaths, is currently not available. Several studies have shown a 10–25% error or discrepancy rate between what clinicians think is the cause of death and the findings of a full traditional autopsy [7, 8], with error rates as high as 50% on medical certificates in stillbirths [9]. These errors may be attributable to the infrequency with which traditional autopsies are performed, and there continues to be little informed support available for parents about autopsy following stillbirth [10].

There is a broad range of perinatal and paediatric diagnoses and causes of death, which are very different from adult mortality causes [11]. This further highlights the need to optimise postmortem examinations to address issues specific to the type of death, which is likely to be encountered in each age or gestational group. These, in turn, need to be carefully formulated into pragmatic clinical guidelines. Some aspects may be intuitive to those medical professionals with a familiarity with this clinical presentation, such as a radiographic skeletal survey being fundamental in paediatric deaths suspicious for inflicted or non-accidental injury.

However, an evidence base is needed to address currently unanswered questions in this population, such as:

- Does a stillbirth with normal antenatal US imaging and normal karyotype benefit from detailed postmortem imaging using CT or MRI, or will other investigations, such as placental histology, provide the likely cause of death in the majority of cases?
- Does conventional postmortem MRI of very small foetuses in the first and early second trimester yield diagnostically useful information?
- In what proportion of cases originally diagnosed as SIDS could postmortem CT or postmortem MRI provide additional clinically relevant information by determining a cause of death?

Investigating these questions may require a coordinated approach between several different centres or different countries to allow sufficient population sampling.

The value of imaging in the paediatric setting should also always be taken in the context of other less-invasive sampling methods, including external examination of the body, skeletal radiographs, photography, pathological assessment of the placenta, noninvasive swabs for microbiology, genetics chromosomal analysis and other biomarkers, as such investigations, traditionally part of the autopsy, often provide additional diagnostic information [11, 12].

### Postmortem imaging in children

Evaluating the true value of postmortem imaging is difficult, but direct comparison between postmortem imaging and autopsy findings have recently been published [11, 13]. In adults, the concordance rates of adult postmortem imaging and full autopsy findings in the only blinded study published to date were disappointing, at around 50% [13]. This included 180 cases, and the major discrepancy rate between cause of death identified by radiology and autopsy was 32% for postmortem CT and 43% for postmortem MRI. Postmortem CT appears to be a more accurate imaging technique than postmortem MRI for providing a cause of death in adults, with a similar error rate to traditional autopsy/clinical death certification. Typical errors included ischaemic heart disease, pulmonary embolism and pneumonia. Whilst there is room for improvement, postmortem CT and postmortem CT angiography (CTA) are already becoming the workhorse imaging modality for adult imaging [14–16].

Recent data confirm that postmortem MRI is likely to become the standard for postmortem imaging in children. A recent benchmark prospective validation study of postmortem imaging in foetuses and children at a specialist children’s hospital in London found a >90% concordance rate between noninvasive postmortem assessment (including postmortem MRI and ancillary investigations not requiring invasive procedures, such as placental examination) and conventional full autopsy findings in 400 cases (277 foetuses, 123 children) [11]. Interestingly, this was greatest for foetuses (<24 weeks 95%, >24 weeks 96%), less good for newborns (81%) and infants (85%), and least good for children ages 1–16 (54%), although these data probably reflect to some extent smaller sample sizes of the latter groups. Based on noninvasive postmortem findings, around 40% of traditional autopsies were judged to be unnecessary, and in these cases there was 99% concordance between conventional autopsy and minimally invasive autopsy. The lower concordance in children was primarily due to undetected infectious pathologies, including pneumonia and myocarditis, for which conventional postmortem MRI currently has a low detection rate [11].

The study also demonstrated that postmortem MRI was particularly useful for intracranial pathology in foetuses, in which the inherent fragility of the foetal brain leads to difficulties with traditional neuropathological examination even following fixation, and in whom adequate pathological examination of the brain may not be possible in around 20% of cases due to changes of autolysis, maceration and artefacts. Of the cases in which intracranial postmortem MRI examination was normal, detailed formal neuropathological examination provided clinically significant new information in less than 1% [11].

Importantly, however, these data also demonstrate that use of postmortem MRI alone, without involvement of a pathologist to perform and interpret ancillary postmortem investigations in conjunction with the clinical features currently has a poor diagnostic yield (around 50%). If this approach is offered in conjunction with noninvasive external examination, then the limitations must be appreciated and adequately explained. Parents should be informed that noninvasive autopsy, including postmortem MRI and ancillary, minimally invasive investigations, performed jointly by a pathologist and radiologist, can in the majority of cases have a similar accuracy to that of conventional autopsy for detection of cause of death and/or major pathology. This approach could therefore allow a triage process in which initial postmortem MRI and other investigations are performed with progression to full or modified autopsy as indicated by the results of the noninvasive postmortem findings. This approach is likely to be useful in improving the uptake of postmortem evaluation for parents in whom current approaches are unacceptable.

### Diagnostic categories/cause of death

**Fig. 2:**
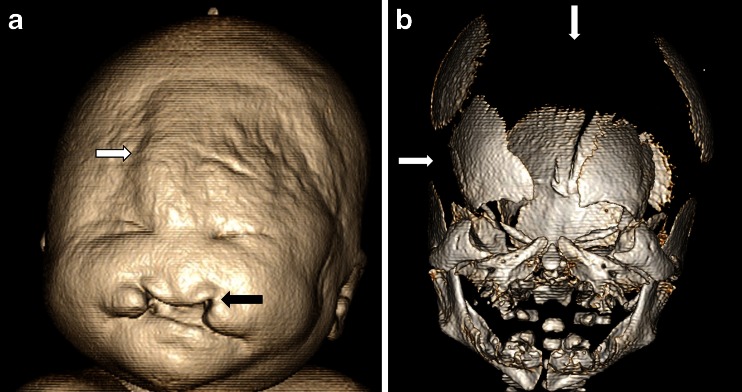
Postmortem CT of a male foetus who died at 22 weeks after a terminated pregnancy following antenatal sonographic diagnosis of holoprosencephaly and a midline facial defect. **a** Surface-rendered postmortem CT shows a cleft lip (*black arrow*), the presence of an interorbital proboscis (*white arrow*) and two eyes. **b** Three-dimensional reconstruction of the skull shows a midline defect with absence of bony medial boundaries of the orbits. Postmortem moulding has caused diastasis of the parietal and occipital bones (*white arrows*). Based on the imaging findings, a diagnosis of ethmocephaly was made; parental consent for autopsy was refused

**Fig. 3:**
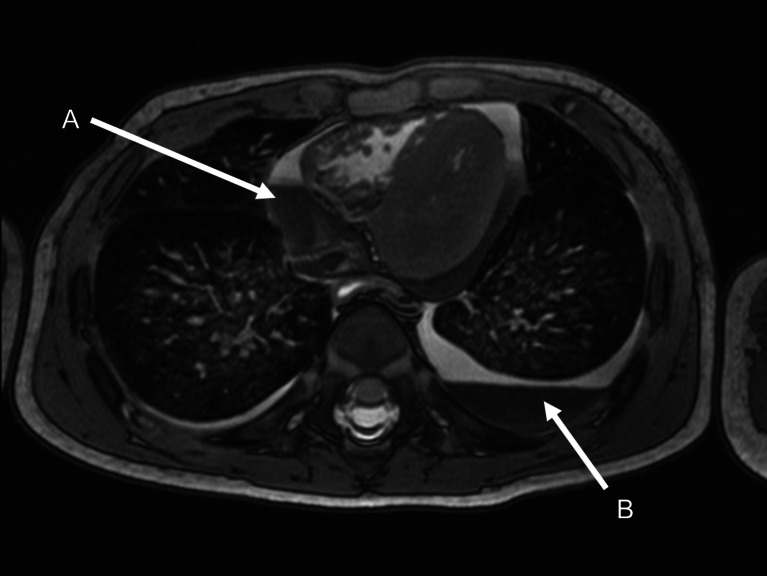
Postmortem axial MRI of the chest of an 8-month-old boy who died of unknown causes. Unusually, there was apparent sedimentation/layering of blood in the pericardial sac (**a**) as well as in the left pleural cavity (**b**), which was attributed to traumatic pericardiocentesis during resuscitation. There was no evidence of trauma

**Fig. 4:**
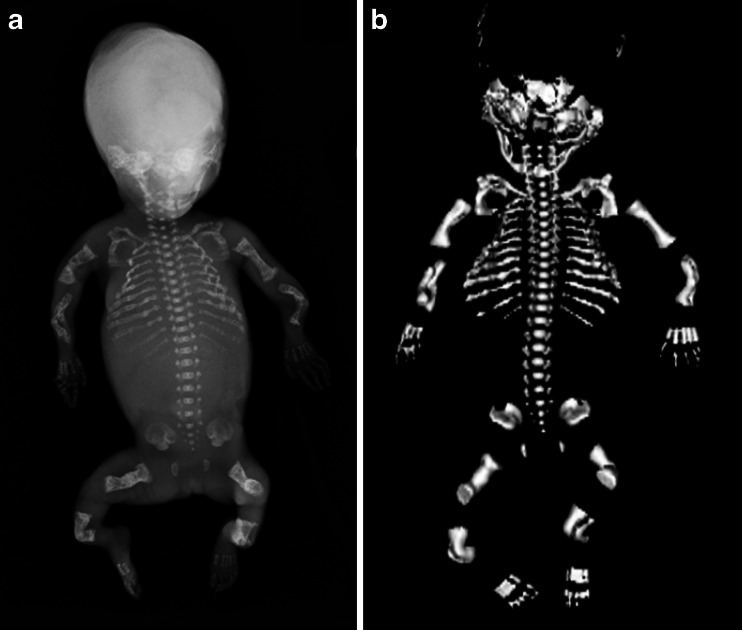
Postmortem skeletal radiograph (**a**) and 3-D postmortem CT reconstruction (**b**) of a 20-week male foetus. The pregnancy was terminated for suspected skeletal dysplasia. There is severe under-mineralisation of the entire skeleton, beading of the ribs and bowing and crumpling of the long bones, caused by multiple fractures. These appearances are typical for lethal congenital osteogenesis imperfecta (type II), which was confirmed on genetic testing

Whilst neurological and cardiovascular disease (such as coronary artery related disease) are likely to account for the vast majority of sudden death in adults, in children the spectrum of disease is markedly different [11, 17]. For foetuses and neonates, congenital abnormalities and complications of delivery account for the majority of deaths, and for foetuses terminated for antenatally detected structural anomalies, genetic and syndromic disorders are the predominant category [18] (Figs. 2,3, and 4). In infancy, the single most common group is sudden and unexpected deaths, often termed SIDS, cot-death or crib-death, in which the mechanism remains uncertain and the diagnosis is one of exclusion following autopsy. Of unexpected infant deaths in which a medical cause is identified, infections, such as unrecognised respiratory tract infection, represent the largest group [19]. Therefore, the optimal strategies for investigating such deaths, including both imaging and ancillary investigations, vary significantly with age. A thorough understanding of the spectrum of likely pathologies is required in order to direct a rational death investigation strategy.

Furthermore, in the paediatric setting, especially foetal and neonatal deaths, there has often been some form of antenatal imaging, usually US. Evaluation of this imaging is useful in several ways, since antenatal findings may be confirmed or refuted on postmortem imaging (such as ventriculomegaly [20]). An individual with no abnormality on either antenatal US or postmortem cross-sectional imaging is unlikely to have a significant anatomical abnormality missed (85% agreement) [21]. Excellent antenatal sonography skills combined with good postmortem imaging may preclude the need for formal invasive autopsy, but this has yet to be fully evaluated.

In some circumstances, histological evaluation of tissue samples is required to determine the cause of death or provide a specific diagnosis and may be the most useful ancillary investigation [22]. In such cases, following postmortem imaging to direct further procedures, tissue may be obtained by either an open or minimally invasive approach, such as the use of endoscopic guided tissue sampling [23], which is likely to represent a more acceptable approach compared to traditional autopsy for many parents [24, 25].

### Skills required to undertake postmortem imaging in children

Just as in adult postmortem imaging, the field of paediatric and perinatal postmortem imaging is highly specialised and relatively new, with relatively few interested and qualified people around the world with the interest and expertise to develop this service further. There is a small but substantial overlap with a range of other fields including forensic imaging (predominantly adult-oriented), paediatric imaging in live children (which share many of the disease and imaging characteristics), and paediatric and perinatal pathology. Perinatal pathology is a separate subspecialty in the United Kingdom with a dedicated training pathway and accreditation.

The skills of a paediatric radiologist, according to the revised European training curriculum for radiology [26], should include:

- an in-depth understanding of developmental anatomy during childhood, a basic understanding of embryology as applied to paediatric diseases,
- an understanding of the various stages of embryonic and foetal development on imaging,
- the ability to describe normal variants in childhood that may mimic disease,
- and familiarity with imaging features of disorders and syndromes in children.

Core radiological skills required include basic anatomy, radiation protection training and physics as applied to several different types of imaging modality. The appropriate use of each imaging modality requires expertise and varies according to the clinical question being addressed and the skill of the radiologist.

Several aspects of foetal development and antenatal imaging are now performed by obstetricians or foetal medicine specialists around the world, as antenatal and paediatric radiology services may be physically separate within a hospital campus. As a result, there is a risk of relatively limited knowledge of foetal imaging among many radiologists, and limited knowledge of MR techniques amongst those who perform antenatal imaging (mostly US). Above and beyond those requirements, forensic imaging requires an in-depth understanding of those cases where the cause of death is uncertain, including suspected inflicted or non-accidental injury, and the concept of SUDI. Forensic imaging also requires a working knowledge of national legal procedures and in-depth knowledge of forensic paediatric medicine, including how to prepare reports and interpret radiological findings for court.

The perinatal pathology skill set has long been recognised to be separate from that in general pathology [27], and now forms its own separate specialty in the United Kingdom. Whilst a common curriculum of basic histopathology training encompassing surgical pathology, autopsy and cytopathology and generic professional discipline are core to any pathologist, subspecialty training in paediatric and perinatal pathology should include:

- specific and detailed knowledge of the pathology and patterns of diseases specific to childhood,
- an understanding of foetal and infant development,
- and familiarity with a wide range of genetic syndromes, prenatal diagnostic techniques and placental pathology.

Training specifically includes the ability to perform autopsies across a range of clinical scenarios including early foetal loss, spontaneous abortion, termination of pregnancy for foetal abnormality, stillbirth, intrapartum death, SUDI and other settings such as specific cardiac, hepatic or metabolic disease. The Royal College of Pathologists (England) states that knowledge of newer radiological techniques such as postmortem MRI, the ability to request radiology appropriate to the case and to appreciate the importance of obtaining expert radiological opinion should be part of the curriculum [28].

Just as paediatric imaging in the living requires a gentle approach in a child-friendly environment to attain maximal success rates, a paediatric postmortem service requires an equally sensitive and ethically sound approach, with an understanding of the specific medicolegal framework in which the work is performed, which may vary with institution, jurisdiction or country.

### Training requirements

The International Society of Forensic Radiology and Imaging (ISFRI) is developing a comprehensive training and accreditation programme for postmortem imaging, predominantly organised around the use of radiographs and postmortem CT and CTA [29]. The recent ISFRI 2013 meeting was dedicated to developing a training strategy for adult postmortem imaging, with focus in five areas including data acquisition, analysis and storage, recommendations for reading/reporting, development of a basic curriculum, certification/revalidation issues, and a collaborative platform for data sharing [30]. All of these issues are equally valid for paediatric postmortem imaging. The need for education has previously been addressed highlighting the specific needs and knowledge base mandatory for reporting postmortem imaging [31].

Furthermore, national reference guidelines are now being written regarding practical postmortem service delivery. Postmortem MRI should be performed within already established centres of specialist paediatric/neonatal pathology, with postmortem MRI carried out by trained MR radiographers, potentially outside normal working hours [32]. In view of the specialist nature of the paediatric examination, those with dedicated specialist imaging skills should perform the assessment. This would be facilitated by centralising services in experienced regional centres. Some of the skills for postmortem MRI would need to be learned through an established national training scheme for paediatric radiologists, and funded research programmes are needed to build the evidence base to determine the types of death in which cross-sectional imaging is an adjunct to, and those in which it can be used as a replacement for, traditional autopsy. Standards of practice will need to be developed, probably establishing the pathologist in a central coordinating role in the investigation of the cause of death, working closely with practitioners who perform and interpret postmortem imaging studies [33].

A training programme dedicated to perinatal and paediatric postmortem imaging interpretation and reporting is clearly needed, given the wide range of congenital and genetic defects commonly seen in the obstetric antenatal screening population, as well as the effects of postmortem changes on imaging appearances. Medical personnel reporting such postmortem imaging must be appropriately trained, irrespective of their background, and will require combinations of skills from different disciplines. These may include common anatomical and radiological issues, including embryology, an extensive knowledge of congenital abnormalities including skeletal dysplasias, imaging techniques and optimisation, and the limitations of each imaging technique, as well as pathological issues, including decomposition, mechanisms of death and the normal range of pathologies that are likely to be encountered.

One limitation to the implementation of postmortem imaging in children is the availability of CT and especially MR scanners. In most hospitals, scanner availability is limited and full to capacity during daytime working hours of clinical imaging for live patients, as would be expected. This implies that, in general, postmortem imaging needs to be performed outside of normal working hours, either in the evenings, at night or on weekends, to fit around conventional clinical lists, although additional costs may be incurred in doing so. To facilitate changes in working practices, collaboration with radiographers or radiological technicians is paramount, to involve them early on in the need for good-quality postmortem imaging.

### Future of postmortem imaging

What needs to be achieved in paediatric and perinatal postmortem imaging in the next decade to establish a working service? Collaborative work within the imaging community is required to optimise imaging protocols and postmortem MRI sequences, with imaging predominantly taking place in specialised centres to a consistently high standard. Clinical guidelines should be developed for the optimal use of different imaging modalities (radiography, US, CT and MRI), such that the most appropriate imaging investigations are carried out according to the clinical scenario. Clarification is required regarding the need for ethical approval or parental consent for imaging investigations, above and beyond that required for clinical assessment. A teaching programme should be developed to help equip those who wish to pursue this subspecialty further with the best current knowledge available from a range of experienced personnel. Ultimately, this will help to better understand difficult cases, such as SUDI — increasing the information collected in this particular cohort of individuals is likely to yield new diagnostic information. Close collaboration among pathologists and radiologists with mutual respect and recognition for different expertise is essential to simultaneously provide a high-quality patient-focused clinical service and avoid turf battles.

## Conclusion

A national and international perinatal and paediatric postmortem imaging service will require a coordinated approach among specialist centres, to share common skill sets and optimise service delivery during the next few years. This may be achieved through the development of an International Paediatric Postmortem Imaging Network or other multi-institutional collaborations.
